# Functional Analysis Using Whole-Genome Sequencing of a Drug-Sensitive *Mycobacterium tuberculosis* Strain from Peru

**DOI:** 10.1128/genomeA.00087-14

**Published:** 2014-02-27

**Authors:** D. Tarazona, V. Borda, M. Galarza, J. C. Agapito, H. Guio

**Affiliations:** a Laboratorio de Biotecnología y Biología Molecular, Instituto Nacional de Salud, Lima, Peru; b Asociación Latinoamericana de Biotecnología (ALBIOTEC), Lima, Peru; c Unidad de Biotecnología Molecular-Laboratorios de Investigación y Desarrollo de Ciência y Tecnología (LID), Universidad Peruana Cayetano Heredia, Lima, Peru

## Abstract

We report the whole-genome sequence of a Latin American-Mediterranean (LAM) lineage drug-sensitive *Mycobacterium tuberculosis* strain from Peru, INS-SEN. The functional analysis revealed more mutations in secondary metabolite biosynthesis, transport, and catabolism (clusters of orthologous groups [COG] category Q) than for other LAM-sensitive strains. This study contributes to the understanding of the genomic diversity of drug-sensitive *M. tuberculosis*.

## GENOME ANNOUNCEMENT

In 2012, there were an estimated 8.6 million new cases of tuberculosis (TB) worldwide. In Peru, the incidence rate for TB was 95 cases/100,000 people, of which 96% of cases were drug-sensitive TB (1). It has been reported that in Peru there is a high diversity of *Mycobacterium tuberculosis* lineages, including Latin American-Mediterranean (LAM) (23.8%), Haarlem (23.8%), T (22.3%), and Beijing (9.3%) (3). We performed whole-genome sequencing and analysis to investigate the genetic diversity and phylogeny relationships of a drug-sensitive strain of *M. tuberculosis*, INS-SEN.

INS-SEN was isolated from Lima, Peru. The establishment of this strain’s lineage was based on 24 mycobacterial interspersed repetitive unit-variable number of tandem repeat (MIRU-VNTR) loci (4) and by single-nucleotide polymorphisms (SNPs) based on phylogeny (5). The genomic DNA of INS-SEN was sequenced to 1,406× coverage, which consisted of 61,422,158 paired-end reads, using the Illumina HiSeq 2000 sequencer machine. Then, the genomic sequence was assembled with BWA v 0.5.9-r16 (6), using the H37Rv genome (AL123456.3) as a reference, producing 18 contigs. The genomic sequence was annotated with the Rapid Annotations using Subsystem Technology (RAST) server (7) and Prokaryotic Genome Annotation Pipeline (PGAAP). A polymorphism study of the INS-SEN genome was carried out by comparative analysis against the genome of the drug-sensitive strain KZN 4207 (LAM lineage) (8) using SNPsFinder (9) to identify the differences between intergenic and coding regions, and then clusters of orthologous groups (COG) (10).

The 24 loci for MIRU-VNTR and SNPs based on phylogeny determined that INS-SEN belongs to the LAM lineage. The genome sequence is about 99.98% completed compared to the H37Rv reference genome, which has a genome size of 4.42 Mb. The INS-SEN strain has a total of 4,383,671 bp, with an average GC content of 65.6%. It contains 4,389 predicted coding sequences (CDSs). A total of 499 polymorphisms were observed in our comparative study, with 440 of these located in the coding regions of the genome that were classified in the following COG categories: secondary metabolite biosynthesis, transport, and catabolism (Q) (*n* = 38); lipid transport and metabolism (I) (*n* = 35); replication, recombination, and repair (L) (*n* = 34); energy production and conversion (C) (*n* = 32); amino acid transport and metabolism (E) (*n* = 31); carbohydrate transport and metabolism (G) (*n* = 27); cell motility (N) (*n* = 26); cell wall/membrane/envelope biogenesis (M) (*n* = 24); coenzyme transport and metabolism (H) (*n* = 23); signal transduction mechanisms (T) (*n* = 21); inorganic ion transport and metabolism (P) (*n* = 21); transcription (K) (*n* = 17); translation, ribosomal structure, and biogenesis (*n* = 14); posttranslational modification, protein turnover, and chaperones (*n* = 13); nucleotide transport and metabolism (*n* = 12); defense mechanisms (*n* = 7); cell cycle control, cell division, and chromosome partitioning (*n* = 7); RNA processing and modification (*n* = 2); and intracellular trafficking, secretion, and vesicular transport (*n* = 2).

INS-SEN had more SNPs in PPE associated with antigenic variation (11) in category N and in PE-PGRS associated with antigenic variation and immune evasion (12) in category M than the strains KZN 4207 and H37Rv. Additionally, INS-SEN showed more mutations in category Q than the strain KZN 4207. It is possible that the organization of SNPs in INS-SEN may have a role in adaptation to its environment.

### Nucleotide sequence accession numbers.

This whole-genome shotgun project has been deposited at DDBJ/EMBL/GenBank under the accession number JAQH00000000. The version described in this paper is JAQH01000000.
